# A Systematic Review of Evaluated Labor Market Initiatives Addressing Precarious Employment: Findings and Public Health Implications

**DOI:** 10.1177/27551938241310120

**Published:** 2025-01-15

**Authors:** Virginia Gunn, Nuria Matilla-Santander, Bertina Kreshpaj, Emilia F. Vignola, David H. Wegman, Christer Hogstedt, Theo Bodin, Emily Q. Ahonen, Sherry Baron, Carles Muntaner, Patricia O’Campo, Wayne Lewchuk, Maria Albin, Kathryn Badarin, Carin Håkansta

**Affiliations:** 1 School of Nursing, 55964 Cape Breton University, Sydney, Nova Scotia, Canada; 2 Unit of Occupational Medicine, Karolinska Institute, Sweden; 3 310844 ISGlobal, Barcelona, Spain; 4 Department of Public Health, 4321 University of Copenhagen, Kobenhavn, Denmark; 5 Epidemiology, 49462 University of Washington School of Public Health, Seattle, USA; 6 14710 University of Massachusetts Lowell, Lowell, USA; 7 Division of Occupational and Environmental Health, Department of Family and Preventive Medicine, 7060 The University of Utah, Salt Lake City, USA; 8 Cuny Graduate School of Public Health and Health Policy, 2009 City University of New York, New York, USA; 9 Barry Commoner Center for Health and the Environment, Queens College, 2009 City University of New York, Flushing, USA; 10 Public Health, Nursing & Psychiatry, 7938 University of Toronto, Toronto, Canada; 11 JHU-UPF Public Policy Center (JHU-UPF PPC), UPF Barcelona School of Management (UPF-BSM), 16770 Universitat Pompeu Fabra, Barcelona, Spain; 12 536977 St Michael's Hospital Centre for Urban Health Solutions, Toronto, Canada; 13 7938 University of Toronto, Toronto, Canada; 14 Faculty of Social Sciences, 3710 McMaster University, Hamilton, Canada

**Keywords:** occupational epidemiology, low-quality employment, non-standard employment, employment quality, social determinants of health inequities, employment insecurity, income inadequacy, lack of rights and protection in the employment relationship

## Abstract

Precarious employment (PE) is a major determinant of population health and contributor to health and social inequities. The purpose of this article is to synthesize and critically appraise available evidence on labor market initiatives addressing PE identified through a systematic review. Of the 21 initiatives reviewed, grouped into four categories—labor market policies, legislation, and reforms; union strategies; apprenticeships and other youth programs; social protection programs—10 showed consistently positive outcomes and 11 a combination of negative, mixed, or inconclusive outcomes. In addition to reviewing the key findings, we discuss public health implications and recommendations related to PE and the implementation and evaluation of initiatives. Given the wide diversity of initiatives, implementation approaches, evaluation methods, and socioeconomic and historical contexts characterizing the labor markets of the countries studied, we refrain from making recommendations regarding the most effective initiatives to address PE. Instead, we discuss several implications concerning the four types of initiatives to further support those searching for solutions to address PE. We strongly recommend tailoring adopted initiatives to local contexts to match a country's specific PE problems and unique labor market and socioeconomic context.

While the construct of precarious employment (PE) continues to undergo refinement, in the public health, social, and occupational epidemiology literature it is framed as a combination of insecurities affecting workers’ employment conditions and income along with diminished control over the work environment, as related to restricted rights and safeguards in the employment relation.^1–4^ PE has gradually become a global phenomenon,^5^ as facilitated by a combination of factors including globalization, neoliberalism, evolving means of production and work patterns, in conjunction with increased worker mobility.^6–8^ Given the compound impacts of PE on workers’ mental^9–11^ and physical health,^12,13^ well-being,^14–16^ lifestyle health behaviors,^17^ occupational health and safety (OHS;^18,19^), and mortality rates,^20^ along with its unequal distribution among population groups,^21–28^ PE is a major determinant of population health (PH) and contributor to health and social inequities.^4,29–33^ Interest in PE and its various manifestations across sectors and occupations may be fueled during economic crises,^34^ including the one triggered by the COVID-19 pandemic,^31,35–39^ due to their detrimental effects on economic growth and the world of work, along with a marked unequal impact on certain worker groups,^40^ especially on large segments of working class occupations.

PE is present across different country-contexts,^6,34,41^ economic sectors,^42,43^ employment circumstances (e.g., platform, informal/undeclared, and nonstandard),^44–47^ and occupations.^11,39,48–50^ Consequently, successful strategies to address PE require tailoring to each specific context. Equally important, given stretched budgets and competing workplace priorities, the selection of PE intervention strategies must consider proven effectiveness as established through evaluations and findings dissemination. While numerous solutions at various levels of action (e.g., individual, community, regional, country, and international) have been proposed to address PE and its impact on workers’ health and well-being,^51–54^ publications describing evaluated interventions are much less common.^9,55,56^

This article synthesizes available evidence on evaluated labor market initiatives addressing workers’ exposure to PE and discusses the key findings considering their PH implications. The focus is on the three dimensions of PE—employment insecurity, income inadequacy, and lack of rights and protection in the employment relation—emphasized in the 2020 Kreshpaj and colleagues synthesis of PE operationalizations^3^ and other multidimensional PE definitions.^1,6,57^ Our review considered all three dimensions of precarious employment (employment insecurity, income inadequacy, and worker rights) when creating the search strategy, establishing the inclusion/exclusion criteria, and describing the precarious employment dimensions targeted by interventions and the outcomes evaluated.

This research is part of a larger systematic review conducted to “identify, appraise, and synthesize existing research on the effectiveness of initiatives aiming to or having the potential to eliminate, reduce, or mitigate workers’ exposure to PE conditions and its effects on the health and well-being of workers and their families”.^56^ Findings from the larger review of interventions addressing PE have been grouped into three separate articles, according to evaluated outcomes, or initiative focus, with each publication covering distinct studies. While this article does not discuss health and well-being outcomes of interventions addressing PE because they were not reported in the included studies, one of the other two studies is dedicated to other initiatives whose impact on worker health and well-being were evaluated and reported (no matter the type of initiatives).^55^ The third article synthesizes evidence regarding minimum wage-related policy initiatives with the potential to address PE.^58^

Given that PE could be a characteristic of both formal and informal work,^45^ although informal work does not contemplate several characteristics of PE (e.g., a labor contract), the initiatives we considered could have targeted formal and/or informal workers, who share a multitude of concerns regarding the stability of their employment, the sufficiency and predictability of their income, and their workplace rights, including the ability to access social protection. Informal work is often described as work that lacks formal employment arrangements and that evades taxation and/or registration by the government.^59^

## Methods

We conducted the review and organized the reporting of methods and findings according to the 2020 PRISMA framework.^60^ A detailed review of the eligibility criteria is listed next and an overview of the search terms, search strategies, languages, and period covered is included in Supplementary Material 1. Further details regarding the planning, structuring, and running of the review are available in the PROSPERO protocol registration^61^ and protocol publication.^56^

### Eligibility Criteria

While this article is dedicated to synthesizing and appraising labor market initiatives addressing PE, the description that follows refers to the approach employed in the conduct of the larger systematic review.

The eligibility criteria were specified according to the following considerations: population of interest, intervention(s) described, outcome(s) evaluated, study design, publication year, and language, as defined next.

Inclusion criteria:

1. Population of interest: workers (18 years of age and older, irrespective of gender, race, ethnicity, and migration status) and workers’ immediate or extended families.
2. Initiatives examined: initiatives that were purposefully designed to address PE or that were designed for other purposes but had the potential to address PE and/or its effects on the health and well-being of workers and their families. Initiatives had to be both implemented and evaluated and were considered regardless of the evaluation results (successful, unsuccessful, or inconclusive). They were defined as broadly as possible and included interventions, policies, legislation/regulations, programs, guidelines, recommendations, collective agreements, and institutional practices.
3. Outcomes evaluated: focused on changes in prevalence of PE, workers’ exposure to PE, or the health and well-being of precariously employed workers and their families.
4. Study design: qualitative, quantitative, or mixed-methods study designs and evaluations.
5. Publication year and language: studies published from January 2000 to May 2021, in any language spoken by members of our review team: Catalan, Danish, Dutch, English, French, Italian, Norwegian, Romanian, Spanish, and Swedish.

Exclusion criteria:

1. Editorial, commentary, discussion paper, review.
2. No clear initiative implemented.
3. Initiative designed to facilitate PE or increase exposure to PE; improve workers’ health through individual behavioral change without a focus on PE; improve work performance or health, safety, or well-being of workers with disabilities without a focus on PE; eliminate or reduce workers’ exposure to unemployment; eliminate, reduce, or mitigate the effects of unemployment on health and well-being; or promote workers’ return to work after illness or injury without addressing PE.
4. Not evaluated formally or assessed using empirical data; the evaluation does not include a clear focus on the reduction of PE and/or on precarious workers and/or their families.
5. Duplicate.
6. Not in a language mentioned in the protocol.

Despite the extensive and systematic search employed to conduct this review (e.g., using a comprehensive search strategy designed in collaboration with two librarians, searching three sources of grey literature and three academic databases determined to have the least overlap; conducting forward and backward citation searches, and asking topic experts for suggestions on relevant studies), it is possible that potentially relevant studies indexed elsewhere or using different key words than those included in the search were missed.

### Quality Appraisal

Because the eligible studies included a combination of qualitative, quantitative, and mixed methods designs we used the Mixed Methods Appraisal Tool (MMAT;^62^) to assess the methodological quality of included studies. This tool evaluates methodological characteristics using three answer options “Yes,” “No,” and “Can’t tell.” Although the tool is not as exhaustive as other tools designed for qualitative and quantitative studies, its personalized questions make it appropriate for various study designs and its established usefulness, reliability, and straightforwardness with various heterogenous studies made it a suitable choice.^63,64^

To support the interpretation of findings, we used the number of “Yes” responses to the screening and quality assessment questions to calculate an overall rating, judging studies with 6–7 “Yes” answers as high quality, studies with 3–5 “Yes” answers as medium quality, and studies with 0–2 “Yes” answers as low quality.

## Results

Twenty-two studies evaluating labor market initiatives addressing PE met the inclusion criteria. An overview of the study identification, screening and inclusion results is provided in Supplementary Material 2.

A high-level synopsis of key characteristics of these 22 studies is provided in Table 1, including the continents represented by the countries examined, the study design, targeted economic sector, categories of labor market strategies, PE dimensions assessed, and quality appraisal rating, with further details provided later in the article.

**Table 1.** Characteristics of the Included Studies.

		Number of studies
**Studies included**		22
**Continents represented by the countries examined** ^a^	Africa	2
	America	10
	Asia	3
	Europe	6
	Oceania	1
**Study design** ^b^	Qualitative studies	1
	Randomized controlled trials	3
	Nonrandomized controlled trials	4
	Quantitative descriptive studies	11
	Mixed-methods studies	3
**Targeted economic sector (ISIC Rev 4)** ^c, d^	All economic sectors	15
	Administrative and support service activities	3
	Manufacturing	2
	Construction	2
	Health and social work	2
	Agriculture, forestry, and fishing	1
	Transportation and storage	1
	Activities of households as employers	1
**Labor market strategies**	Labor market policies, legislation, and reforms	10
	Union strategies	2
	Apprenticeships and/or other programs focused on youth and new graduates	7
	Social protection programs	3
**Initiative being purposefully designed to address PE**	No	16
	Yes	6
**PE outcomes evaluated** ^d^	Employment insecurity	19
	Income inadequacy	5
	Lack of rights and protection in the employment relation	3
**Quality appraisal rating** ^e^	Low quality (0 to 2)	0
	Medium quality (3 to 5)	6
	High quality (6 to 7)	16

Notes:

^a^ The grouping was done using the World Health Organization regions.

^b^ This categorization of study design uses the categories included in the Mixed Methods Appraisal Tool (MMAT), 2018 version.

^c^ Classification based on United Nation's International Standard Industrial Classification of all Economic Activities (ISIC) Rev.4 available via https://ilostat.ilo.org/resources/concepts-and-definitions/classification-economic-activities/

^d^ The sum could be more than 22 given that several studies targeted several economic sectors and evaluated several PE outcomes.

^e^ Quality appraisal rating interpretation: To calculate the rating, we used the number of “yes” responses to the quality assessment questions included in the MMAT 2018 version, including the two screening questions. Low quality (0–2 “yes” answers), medium quality (3–5 “yes” answers), and high quality (6–7 “yes” answers).

We grouped the 22 studies, evaluating 21 initiatives (two studies evaluated the same initiative^65,66^), into four categories of labor market initiatives: labor market policies, legislation, and reforms; union strategies; apprenticeships and/or other programs focused on youth and new graduates; and social protection programs, as presented in Table 2.

**Table 2.** Initiatives, Ways in Which They Could Impact Precarious Employment (PE), PE Outcomes Evaluated, Evaluation Results, and Quality Appraisal Rating.

Study author(s) Publication year Countries examined	Implemented initiatives	Specific dimension(s) of PE potentially impacted	Ways in which the initiative could impact PE	PE outcomes evaluated and evaluation results	Quality appraisal rating*
**Labor market policies, legislation, and reforms**					
Fagernas, 2010 India	State-level changes to industrial labor disputes legislation and the dispute settlement process to strengthen job security and protect the rights of workers and employers in labor disputes.	Employment insecurity Income inadequacy Lack of rights and protection	Eliminate, reduce, or mitigate workers’ exposure to PE	**Employment insecurity** – No clear relationship found between labor regulation or the dispute-settlement process and the share of permanent salaried workers and temporary workers. The results could not confirm a speculated negative relationship between pro-worker regulation and the share of permanent salaried workers. INCONCLUSIVE	High
Pla-Julián, 2014 Spain	Law reforms concerning household employment, aimed at improving the employment and working conditions of domestic workers.	Employment insecurity Income inadequacy Lack of rights and protection	Eliminate, reduce, and mitigate workers’ exposure to precarious and informal employment	**Employment insecurity** – The reforms were linked to the formalization of the employment relationship and a slight reduction in the overall degree of worker informality. POSITIVE **Income inadequacy** – Some workers experienced a wage reduction due to formalization-related costs. MIXED **Lack of rights and protection** – A relative increase was observed regarding the number of domestic workers eligible for the social security system in the short-term (follow-up of about 1–2 years). POSITIVE	High
Arranz et al., 2013 Spain	Adoption of active labor market policies, such as employment subsidies for permanent contracts, job-creation schemes, and vocational training programmes to fund/incentivize national-level job creation and permanent employment over a period of 24 years.	Employment insecurity	Eliminate and reduce workers’ exposure to PE	**Employment insecurity** – Employer subsidies for permanent contracts were estimated to have a small positive impact on transitions from temporary to permanent employment. However, the subsidies did not have a notable impact on the aggregate levels of permanent or temporary employment. Job-creation programs and training had limited effects on the creation of permanent contracts. MIXED	Medium
Mendez, 2013 Spain	Implementation of two major labor market reforms in Spain in 1994 and 1997 to reduce temporary employment and promote permanent employment.	Employment insecurity	Eliminate and reduce workers’ exposure to PE	**Employment insecurity** – Both reforms failed to (*a*) convert temporary contracts into permanent ones and (*b*) increase the use of permanent contracts. Although employers hired workers into permanent contracts, they also substituted permanent contracts for temporary ones, exploiting the lowering of dismissal costs. The limitations on the use of non-causal temporary contracts triggered the use of other types of temporary contracts, instead of boosting the use of permanent contracts. NEGATIVE	Medium
Ciani and de Blasio, 2015 Italy	Use of a national program offering incentives to stimulate employers to convert fixed-term contracts into open-ended ones.	Employment insecurity	Reduce workers’ exposure to PE	**Employment insecurity** – The use of incentives is estimated to have increased the probability of converting fixed-term contracts into open-ended ones by 83%, with larger effects observed for men < 30 years old and women > 30, and smaller effects for younger women. 90% of all incentives were distributed for conversions or stabilizations of temporary contracts. No evidence was found that employers delayed conversions after being funded or reduced the conversion rate after the exhaustion of funds. POSITIVE	Medium
Borooah et al., 2007 India	Adoption of jobs reservation policies (affirmative action programs) to reserve a proportion of government or public sector jobs for persons from certain Scheduled Castes and Scheduled Tribes.	Employment insecurity	Reduce workers’ exposure to PE	**Employment insecurity** – The job reservation system increased regular salaried and wage employment* amongst disadvantaged groups by about 5 percentage points. *Regular salaried or wage employment is viewed as being stable and secure, with decent income, meeting labor standards and providing social protection. (Fields, 2011) POSITIVE	High
Giovannetti et al., 2021 Egypt	Adoption of tariff reforms (protectionist policies) to protect certain economic sectors from foreign competition, with a focus on protecting wages and job stability, including having a permanent position.	Employment insecurity	Reduce workers’ exposure to PE	**Employment insecurity** – Results suggest that trade tariffs are negatively correlated with a worker's probability of having a permanent contract, compared to the probability of having a casual, temporary, or seasonal contract. Tariff liberalization does not seem to negatively impact job stability. NEGATIVE	High
Selwaness and Zaki, 2015 Egypt	Adoption of trade liberalization reforms consisting of tariff reductions leading to lower trade costs (with potential to decrease informal employment).	Employment insecurity	Reduce workers’ exposure to informal employment	**Employment insecurity** – The reduction of tariffs resulted in a slight reduction in informal employment in the manufacturing sector. Given that women form a large share of informal employment, it is likely that this initiative is particularly beneficial for them. POSITIVE	High
Rothenberg et al., 2016 Indonesia	Introduction of “one-stop-shops” for business registration, a large-scale program attempting to reduce registration costs for businesses, with the goal of reducing the number of informal businesses.	Employment insecurity	Reduce workers’ exposure to informal employment	**Employment insecurity** – The initiative did not reduce workers’ probability of being informally employed and did not facilitate their shift from informal to formal employment. NEGATIVE	High
Osorio-Copete, 2016 Colombia	Adoption of a tax reform lowering costs for firms with the goal of promoting labor formalization.	Employment insecurity	Reduce workers’ exposure to informal employment	**Employment insecurity** – In the long-term, the new tax reduced labor informality by 2.3 percentage points, although the initial, short-term effects of the tax reform indicated an increase in labor informality. MIXED	Medium
**Union strategies**					
Wright, 2013 UK	Adoption of two strategies (community unionism, and sustainable sourcing) by unions to reach precarious workers.	Employment insecurity Income inadequacy Lack of rights and protection	Eliminate, reduce, and mitigate workers’ exposure to PE	**Reaching workers in PE** – Both strategies present reasonably effective ways of reaching precarious workers. While the study did not evaluate the direct impact of these strategies on the precarious workers engaged by them, (*a*) a decline in union strength has been linked to an increase in the number of workers in PE; and (*b*) being unionized, and benefitting from union support has been linked to improved employment and working conditions for workers. INCONCLUSIVE	High
Theodore, 2020 US	Use of worker centers for the hiring of day labors instead of them being hired at informal hiring sites (such as street corners).	Employment insecurity Income inadequacy Lack of rights and protection	Reduce and mitigate workers’ exposure to precarious and informal employment	**Income inadequacy** – Higher average wages for workers using worker centres when compared to those using informal hiring centres. The setting of occupation-specific minimum wages by worker centres facilitated higher wages for workers using them. While the 2012 data showed a positive impact for workers in less-skilled jobs, such as moving and cleaning, the 2015 data suggested that workers in higher paying jobs also benefitted from the use of workers centres. POSITIVE **Lack of rights and protection** – Lower levels of wage theft for workers using the worker centres when compared to those using informal hiring centres. POSITIVE	Medium
**Apprenticeships and other programs focused on youth and new graduates**					
Baumann et al., 2013 Canada	Adoption of a governmental policy initiative to encourage full-time employment for new nurse graduates through the provision of funding (up to six months for salary and benefits) to employers hiring nurse graduates in full-time nursing positions, with the expectation that the full-time employment will be extended after the funded period.	Employment insecurity	Reduce workers’ exposure to PE	**Employment insecurity** – New graduate nurses participating in the initiative in 2012–2013 secured permanent full-time employment at higher rates than those who did not participate (62% of registered nurses and 50% of registered practical nurses who participated vs. 38% and 17% who did not, respectively). POSITIVE	High
Baumann et al., 2012 Canada	Same initiative as the one described in the study (Baumann et al., 2013) above.	Employment insecurity	Reduce workers’ exposure to PE	**Employment insecurity** – Over the period assessed, a trend that had previously more nurse graduates in part-time work reversed, showing the majority in full-time employment. The increase in full-time employment was found for all health sectors, (12 to 24% for new registered nurses and 15 to 31% for new registered practical nurses). POSITIVE	High
Albanese et al., 2021 Italy	Reform of a national apprenticeship system to facilitate stronger links between apprentices and employers and reduce bureaucracy facing companies offering the training.	Employment insecurity Income inadequacy	Reduce workers’ exposure to PE	**Employment insecurity** – The reform increased the rate of transition to permanent jobs, with the strongest effects observed in companies with more than 10 employees (the rate increased by 39.7% within the same firm after four years). POSITIVE **Income inadequacy** – Sizeable initial positive effects on wages, with reformed wages almost 20% higher. While the wage gap diminished in the first two years, a positive long-term effect was found (+7%), extending well beyond the legal duration of the apprenticeship contract, and consistent with the pattern of higher job stability. POSITIVE	High
Corseuil et al., 2019 Brazil	A national apprenticeship program, consisting of special contracts providing classroom and skill training to young workers.	Employment insecurity	Mitigate workers’ exposure to PE	**Employment insecurity** – The program increased the probability of employment in an open-ended formal job by 7.9% after 2–3 years and by 6.9% after 4–5 years, relative to other temporary contracts. POSITIVE	High
Ibarrarán et al., 2019 Dominican Republic	Offer of a paid job-training program consisting of classroom and on-the-job training along with an internship for disadvantaged youth.	Employment insecurity Income inadequacy	Reduce workers’ exposure to precarious and informal employment	**Employment insecurity** – The program had significant and positive effects on the probability of being formally employed, particularly for men. POSITIVE **Income inadequacy** – The program had positive effects on earnings, especially for young women in key labor market contexts, such as the capital and the regions surrounding it, probably linked to a higher demand in those regions for the skills acquired through the program. POSITIVE	High
Calero et al., 2017 Brazil	Provision of arts-based interventions (arts- and theater-based pedagogic tools) to youth from disadvantaged socioeconomic contexts, part of a larger training program that includes vocational and academic training along with training in work-readiness skills.	Employment insecurity	Reduce workers’ exposure to precarious and informal employment	**Employment insecurity** – The results show no impact on the formality of jobs held by participating youth in the first 13 months after program completion. INCONCLUSIVE	High
Jackson and Collings, 2018 Australia	Completion of two types of practical experiences (unpaid work-integrated learning and paid work) by university students during their final year of studies.	Employment insecurity	Reduce workers’ exposure to PE	**Employment insecurity** – While participating in work-integrated learning did not impact full-time employment rates, engagement in paid work during the final year of study was linked to a considerably higher likelihood of obtaining both short- and long-term full-time employment. MIXED	High
**Social protection programs**					
Saavedra-Caballero and Londoño, 2018 Colombia	Temporary provision of conditional cash transfers to low-income or informally employed individuals and families with children aged 0 to 17.	Employment instability Lack of rights and protection	Mitigate workers and their families’ exposure to precarious and informal employment	**Employment insecurity** – The findings suggest that receiving the conditional cash transfer increases the probability of being in informal employment in the short-run. NEGATIVE **Lack of rights and protection** – The program (*a*) decreased the probability of not being enrolled in social security in the short term, with no effect observed in the medium/long term; and (*b*) increased the probability of being enrolled in a subsidized health system compared to being enrolled in “contributory health services” in medium/long run. MIXED	High
Thornton et al., 2010 Nicaragua	Provision of a subsidized voluntary health insurance program to informal sector workers.	Lack of rights and protection	Mitigate workers and their families’ exposure to informal employment	**Lack of rights and protection** – Low program enrollment (20% of eligible workers) and low retention rates after the subsidy expiration (less than 10% of those in the program still enrolled after one year). No evidence of significant increase in health-care utilisation among the newly insured. Out-of-pocket expenditures decreased, although the average resulting savings were smaller than the price of the insurance premiums. MIXED	High
Azuara and Marinescu, 2013 Mexico	Provision of public health coverage to uninsured workers, such as informal salaried workers and self-employed workers, and individuals not economically active.	Lack of rights and protection	Mitigate workers and their families’ exposure to informal employment	**Employment insecurity** – The provision of public health coverage had no effect on the level of informality in the employed population. The program was associated with an increase in informality among low-educated workers, but the effect was small (1.7%) and did not increase over time. POSITIVE **Income inadequacy** – The wage improvements for workers moving between the formal and informal sectors were not significantly impacted, implying that marginal workers (defined as “workers who are close to indifferent between working in the informal and formal sector”) do not select between formal and informal jobs based on the availability or cost of health insurance. INCONCLUSIVE	Medium

Notes: *Quality appraisal rating interpretation: To calculate the rating, we used the number of “Yes” responses to the quality assessment questions included in the MMAT 2018 version, including the two screening questions. Low quality (0–2 “Yes” answers), Medium quality (3–5 “Yes” answers), and High quality (6–7 “Yes” answers). Given that we did not deduct points for “Can’t tell” answers (indicating insufficient details available to appraise all methodological aspects), it is possible that our overall rating is more favourable than it would have been if ‘Can’t tell’ answers were accounted for.

Based on their scope, of the 21 initiatives, only four^67–70^ had the potential to influence all three PE dimensions—employment insecurity, income inadequacy, and lack of rights and protection in the employment relation—highlighted in the 2020 Kreshpaj and colleagues review and synthesis of PE operationalizations^3^ and other multidimensional definitions.^1,6,57^ Three initiatives had the potential to affect two dimensions—employment insecurity and lack of rights and protection,^71^ or employment insecurity and income inadequacy^72,73^—while 15 initiatives could have impacted one dimension only, either employment insecurity^65,66,74–84^ or the lack of rights and protection.^85,86^

To support the interpretation of findings in light of their methodological quality, we included the appraisal rating we calculated for each study in Table 2, along with the overview of evaluated initiatives and outcomes. Details regarding the quality assessment conducted using the MMAT 2018 version^62^ are included in Table 3. This table displays the appraisal results regarding clarity of research questions and appropriateness of research approach, data collection methods, analysis, and findings for each study. Studies are grouped by study design, which varied, including one qualitative study,^67^ three randomized controlled trials,^73,84,87^ four nonrandomized quantitative studies,^74,79,83,85^ 11 quantitative descriptive studies,^65,69–72,75–77,80–82^ and three mixed-methods studies.^66,68,78^

**Table 3.**
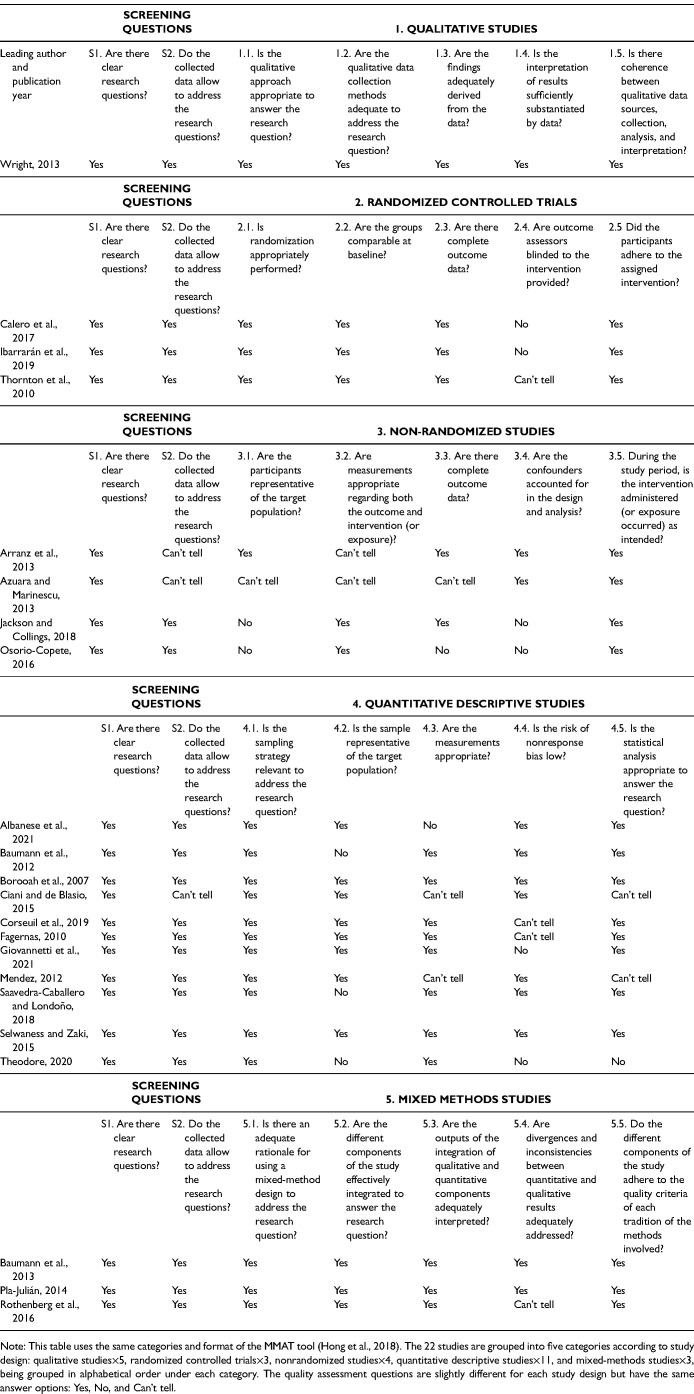
Critical Appraisal of Included Studies Using the Mixed Methods Appraisal Tool (MMAT).

Overall, the quality appraisal ratings were positive, indicating a well-founded body of evidence. There are several key strengths across the 22 included studies. All studies posed clear research questions and all, except three where the reviewers could not tell,^74,77,85^ collected data well suited to address the research questions. All but two^79,83^ quantitative studies had suitable sampling approaches to address the research questions and all except two^65,71^ had samples representative of the target populations. Randomization was appropriately performed for all three randomized controlled trials. Key weaknesses included not accounting for confounders in two^79,83^ of the four nonrandomized controlled trials; inappropriate measurements in one^72^ of the 15 quantitative descriptive or nonrandomized studies and insufficient information to answer this question in four of them;^74,75,77,85^ and a negative assessment on the question whether the outcome assessors were blinded to the intervention for two^73,84^ of the three randomized controlled trials, and insufficient information about blinding for the third one.^87^

Details regarding study design and data collection/analysis approaches employed in each study are included in Supplementary Material 3, along with further details about study objectives, implemented initiatives, economic sector and population subgroups targeted. Specifics regarding study design and data collection/analysis approaches are meant to enable the interpretation of each study's findings in light of methodological aspects such as sources of data, years covered, recruitment and sampling choices, analyses performed, and evaluation strategies. Overall, included studies employed (*a*) rich data sources consisting of national population-level statistics, labor force, and labor market surveys; (*b*) extended longitudinal and cross-sectional analyses; and (*c*) quasi-experimental designs, and other design or estimation approaches (e.g., difference-in-difference) to overcome data availability limitations.

### Labor Market Policies, Legislation, and Reforms

Ten studies evaluated a range of labor market policies, legislation, and reforms, with a combination of positive,^68,76,77,80^ negative,^75,78,81^ inconclusive,^69^ or mixed^68,74,79^ outcomes for several dimensions of PE. Two initiatives attempted to strengthen job security, ensure adequate pay, and enhance workers’ rights and protections through legislation, including changes to industrial labor disputes legislation and the dispute settlement process^69^ and reform of household employment laws.^68^ Three initiatives aimed to stimulate permanent employment and reduce temporary contracts through financial incentives such as subsidies,^77^ reduced social security contributions,^74,75^ and tax reductions;^75^ reduced regulations (e.g., decreased notice period before firing, lowered severance pay);^74,75^ and provision of vocational training programs to expand and update workers’ skills and retrain individuals.^74^ One initiative intended to increase job security by reserving a proportion of government and public sector jobs—viewed as stable and secure^88^—for representatives of groups who have historically been denied access to such jobs due to class and religious discrimination.^76^ Another initiative aimed to increase job stability through the adoption of protectionist tariff reforms shielding certain economic sectors from foreign competition.^81^ Three initiatives intended to reduce labor informality through strategies to reduce business costs to companies: trade liberalization reforms consisting of tariff reductions to lower trade costs,^80^ streamlining business registration processes,^78^ and reforming taxes.^79^

### Union Strategies

Two studies evaluated union strategies. One examined unions’ adoption of community unionism and sustainable sourcing to reach precarious workers.^67^ Community unionism aims to organize precarious workers outside of the workplace, based on the premise that traditional workplace-based strategies used by unions are not well suited for reaching and organizing workers in nonstandard employment. Sustainable sourcing suggests that, when negotiating contracts with suppliers and subcontractors, large firms and public entities should prioritize compliance with labor standards and the extension of collective agreements, and other strategies to improve working conditions, to all workers employed by suppliers and subcontractors, including those in nonstandard employment. Both strategies present reasonably effective ways of reaching workers in PE. However, the study did not evaluate the direct impact of these strategies on PE, focusing instead on process indicators. The second study showed that the use of worker centers to hire day laborers instead of informal hiring sites such as street corners had positive outcomes for both workers’ income and their rights and protections.^70^

### Apprenticeships and Other Programs Focused on Youth and New Graduates

Seven studies examined the effect of apprenticeships and other programs focused on youth and new graduates, with predominately positive effects on employment insecurity^65,66,72,73,82^ and income inadequacy,^72,73^ though inconclusive^84^ and mixed^83^ effects were also reported. One initiative consisted of a governmental policy to stimulate full-time employment for new graduates by providing funding to employers with the expectation that full-time employment will be extended after the funded period.^65,66^ Two initiatives centered on national apprenticeship programs^72,82^ and three on a combination of vocational and academic training,^73,83,84^ all with the goal of increasing youth's work-readiness skills. Except for the provision of arts-based interventions and completion of practical work-related experiences by university students, which showed inconclusive and respectively mixed effects, all other initiatives showed positive results.

### Social Protection Programs

The three social protection programs that were evaluated consisted of the provision of temporary conditional cash transfers to low-income or informally employed individuals and families with children,^71^ a subsidized voluntary health insurance program to informal sector workers,^87^ and public health coverage to uninsured workers (e.g., informal salaried workers and self-employed workers) and individuals not economically active.^85^ The evaluation results were mixed. For instance, while in the short-term the provision of conditional cash transfers increased employment insecurity by increasing the probability of being in informal employment,^71^ the provision of public health coverage had no effect on the level of informality in the employed population.^85^ Regarding the lack of rights and protections dimension of PE, the provision of conditional cash increased workers’ probability of being enrolled in a subsidized health system,^71^ and the provision of subsidized voluntary health insurance decreased out-of-pocket expenses.^87^

## Discussion

### Labor Market Initiatives Addressing Precarious Employment

Given the complex and varied forms of PE, the diversity of available solutions and the ways in which they could impact PE is not surprising. As evident in the review's findings, a wide range of labor market initiatives, including labor market policies and legislation, union strategies, apprenticeships and other programs focused on youth, and social protection programs, have been adopted with varying degrees of success to improve the employment and working conditions of workers. While only five of the 22 initiatives were purposefully designed to address PE,^68,69,74,75,77^ all of them had potential to affect one or more of its dimensions. Further, while most initiatives were designed to act at a macro-level (e.g., national or provincial/state policies), five consisted of organizational or community-level interventions,^67,70,73,77,85^ and two targeted individuals and/or small groups of workers.^83,84^ Considering the diversity of initiatives, implementation approaches, evaluation methods, and socioeconomic and historical contexts characterizing the labor markets of the countries studied, we refrain from making recommendations regarding the most effective initiatives to address PE. Instead, in addition to synthesizing the findings, we discuss several specific considerations and critiques concerning the four types of initiatives to further support those searching for solutions to address PE.

Efforts to improve employment and working conditions often target a country's labor market policies and legislative framework due to the significant influence of these structural factors on employment quality. In some instances, policy and regulatory changes aimed to enhance employment security directly by promoting permanent contracts and reducing temporary ones^74,75,77^ while, in others, the policies aimed to restrict employment in the informal economy.^78,79^ Further, in some cases, the legislation adopted is meant to shield workers and the quality of their employment from the possible damaging effects of globalization or increased international competition.^81^ In others it is meant to liberalize trade to promote economic growth and create employment,^80,89^ although these initiatives are often found to decrease secure employment and incomes, instead of increasing them. While these two strategies—one aimed at restricting trade, the other aimed at expanding it—have the potential to address employment insecurity and income inadequacy, they reflect opposing political ideologies vis-à-vis determinants of economic prosperity and subsequent worker well-being. Further, some policy changes aimed to improve employment and working conditions for workers in a given sector^68^ or for certain groups of workers, such as those who have been marginalized historically, and who suffered negative health, cultural, political, and economic implications on the basis of race, ethnicity, religion,^76^ gender, age,^82^ or sexual orientation, to name only a few.

Union membership and collective bargaining are widely used strategies to enhance workers’ employment and working conditions,^90^ often with positive impacts on workers’ health and well-being.^91–94^ Conversely, the gradual decline in union presence and bargaining power in recent decades has been linked to a worsening of employment and working conditions, and reduced workers’ rights and social protection.^95,96^ One of the two evaluated union initiatives in our review exemplifies the collective efforts to improve employment and working conditions for workers in PE undertaken by self-organized worker groups in countries with weak trade unions.^70^ Yet, even in countries with a strong union presence, workers in nonstandard and informal employment are often excluded from the positive influence of unions for two reasons: their employment arrangements and informality make them difficult to reach; or they are denied union membership based on commonly held beliefs that workers in PE could endanger the employment conditions of other union members by legitimizing nonstandard and precarious forms of employment.^67,97^ There are, however, several examples of positive effects of union membership on health and well-being among PE workers.^93,94^ The recognition that PE is on the rise has intensified unions’ efforts to extend their membership to include workers in PE,^67,98^ which could also serve to increase unions’ capacity to strengthen their membership more generally and reinforce their legitimacy to the larger community.^70,99^ In countries with a strong union presence, where both employers and employees are highly organized (e.g., Scandinavian countries), the responsibility for implementing strategies to improve OHS and other employment and working conditions may rest heavily on the social partners (i.e., employee and employer representatives), which influences the preferred level of action (e.g., agency versus more detailed legislation/state intervention).^44^

Apprenticeships and other programs focused on youth and new graduates are typically based on the premise that young workers face challenges when transitioning from education to work, related to lack of social capital and job-ready skills, along with reluctance of employers to hire young workers^72,73,82^ and, as a result, may be pushed into PE. Common strategies include regulation to promote hiring youth in full-time employment^65,66^ or the provision of apprenticeships to improve job-ready skills and provide temporary jobs as a stepping stone to permanent employment,^72,73^ with the underlying criticism, however, that such temporary jobs could lead youth into a cycle of PE.^82^

The provision of social protection programs is meant to mitigate PE workers’ lack of benefits, such as access to unemployment insurance and subsidized health and social services.^52,100^ Social protection schemes could help individuals avoid poverty traps and material deprivation, commonly found among those in PE,^71,101^ and could both increase health care utilization and minimize related costs.^85,87^ However, if such programs are means-tested (e.g., based on criteria such as being in informal work or a low income) instead of being universally provided, some argue they can create incentives for people to maintain their status quo—for example, staying in a certain type of employment that qualifies them for that social protection program^71,85^ and thus enforcing the cycle of PE and poverty traps. Programs aimed at reducing poverty via means-tested programs have the paradoxical effect of maintaining inequality and poverty.^102^

Future actions addressing PE should consider not only whether prior initiatives were successful, but the reasons contributing to their success or failure. These include unique labor market and socioeconomic contexts; implementation and enforcement approaches, including possible loopholes exploited by employers; and evaluation approaches, methodology, and timing, with consideration for delayed effects of interventions and/or change in direction of effects.

### Public Health Implications and Recommendations

PE is a significant determinant of PH and social inequalities;^4,29–33^ it is, therefore, imperative that PH professionals take an active role in addressing it. To that end, eliminating, reducing, or mitigating workers’ exposure to PE conditions should be recognized as a standard component of public health programing, similar to other standards such as chronic disease prevention, food safety, immunization, school health, infectious and communicable diseases prevention and control, and healthy environments. Doing so will also legitimize PE as a PH concern that public officials and politicians are accountable to respond to through funding allocation. It will also likely increase the predictability and consistency of funding, so that sufficient funds, efforts, and skilled human resources are allocated to both assess PE's population health impacts and design and collect indicators and surveys that can adequately monitor its distribution among the working population and evolution over time. Such efforts should include agreeing on common theoretical frameworks and operationalizations, and incorporating indicators of employment quality, including PE, in health surveillance and labor force surveys to strengthen information systems monitoring PE.^8,101,103^ An ability to more accurately track the prevalence of PE and examine its relationship with health indicators will justify a greater focus within PH on this topic. Further, greater investment in evaluation of PE initiatives will allow evaluation plans to be devised and baseline indicators to be collected before an initiative is implemented, leading to a better understanding of its impact. Many evaluations are conducted retrospectively, using readily available indicators that have been collected for other purposes and that are therefore not necessarily the most appropriate to assess PE. This approach makes it more difficult to understand the context in which an initiative was implemented and its impact on various PE dimensions for different worker groups, which, in turn, limits generalizability of findings to other contexts. To address existing limitations in the current body of evidence examining effectiveness of initiatives addressing precarious employment, future research on this topic should be considerably expanded to increase the number and type of initiatives evaluated, to diversify the sample populations and indicators (both explanatory and outcome) studied, and strengthen research designs. A wide range of longitudinal, cross-sectional, experimental, and observational study designs are needed, using both quantitative, qualitative, and mixed-methods data collection and analysis approaches, and involving those affected in the creation of knowledge, to gain a thorough understanding of the effectiveness of interventions targeting precarious employment.

Recognition of PE as a PH problem has the potential to increase cross-disciplinary collaborations between PH professionals, PH researchers, epidemiologists, economists, and other social scientists, OHS specialists, union representatives, organizational leadership teams, policy analysts, politicians, and other decision makers. In turn, enhanced cross-disciplinary collaborations will inform the creation of comprehensive social protection, economic and labor market initiatives, and policies with potential to eliminate, minimize, or mitigate exposure to PE. The nuanced insights into PE gained by researchers working in different fields will help expand the repertoire of evaluated initiatives and fill existing gaps. For instance, we are aware of the use of public procurement by governments as a strategy to enhance suppliers and subcontractors’ adherence to labor standards and legislation meant to improve OSH and employment and working conditions, but we could not find any initiatives evaluating its use. Additionally, acknowledging PE as a major determinant of PH and social inequities will further justify the need for “health in all” policy approaches, which integrate health considerations across economic sectors.^104^ Another consideration is the rapid transformation of labor markets, precipitated in recent years by the green energy transition and its push for economic systems that favor the reuse and regeneration of materials or products, which is likely to amplify the mismatch between available jobs and the current workforce's skills and competencies, and increase competition for jobs in the low-skilled end of the labor market.^7^ This hasty labor market transformation highlights the urgency of adopting policies that enable sustainable and inclusive growth,^7^ including by addressing PE at different levels.

The involvement of PH researchers will increase the likelihood that planned strategies target not only PE but also its health outcomes, a focus largely missing in existing initiatives.^55^ Further, the increased involvement of PH professionals and researchers will likely prompt the collection of more health and social indicators and an increased evaluation focus on health and social aspects, which is scarce in current evaluations of initiatives addressing PE.^55^ Similarly, the focus on increased health and social inequities in relation to PE and on understanding the ways in which PE affects discriminated population groups (e.g., women, migrants, racialized individuals, etc.) differently will also increase.^8^

In summary, advances in research and advocacy resulting from a wide recognition of PE as a PH problem would lead to health and employment-related interventions, including policy changes, that could ultimately help reduce health inequalities and improve quality of life among working class communities.

## Conclusion

In this article we synthesized available evidence on labor market initiatives addressing precarious employment identified through a systematic review. Of the 21 initiatives reviewed, grouped into four categories—labor market policies, legislation, and reforms; union strategies; apprenticeships and other youth programs; and social protection programs—10 showed consistently positive outcomes and 11 a combination of negative, mixed, or inconclusive outcomes. Given the wide diversity of initiatives, implementation approaches, evaluation methods, and socioeconomic and historical contexts characterizing the labor markets of the countries studied, we refrain from making recommendations regarding the most effective initiatives to address precarious employment. Instead, we discuss several implications concerning the four types of initiatives to further support those searching for solutions to address precarious employment.

While there is a vast body of research examining the numerous ways in which precarious employment affects the health and well-being of workers in specific sectors, precarious employment is not a problem limited to certain sectors, occupations, and forms of employment. For this reason, while tailoring precarious employment initiatives to each context is important, we should not limit our efforts to solutions that address the employment and working conditions of certain worker groups or specific occupations and/or industries. Instead, by acknowledging precarious employment as a determinant of public heallth, we could plan and implement higher-level solutions that act on all precarious employment dimensions and impact more efficiently and sustainably a multitude of health statuses and problems at a population level.

## Supplemental Material

sj-docx-1-joh-10.1177_27551938241310120 - Supplemental material for A Systematic Review of Evaluated Labor Market Initiatives Addressing Precarious Employment: Findings and Public Health Implications Supplemental material, sj-docx-1-joh-10.1177_27551938241310120 for A Systematic Review of Evaluated Labor Market Initiatives Addressing Precarious Employment: Findings and Public Health Implications

sj-docx-2-joh-10.1177_27551938241310120 - Supplemental material for A Systematic Review of Evaluated Labor Market Initiatives Addressing Precarious Employment: Findings and Public Health Implications Supplemental material, sj-docx-2-joh-10.1177_27551938241310120 for A Systematic Review of Evaluated Labor Market Initiatives Addressing Precarious Employment: Findings and Public Health Implications

sj-docx-3-joh-10.1177_27551938241310120 - Supplemental material for A Systematic Review of Evaluated Labor Market Initiatives Addressing Precarious Employment: Findings and Public Health Implications Supplemental material, sj-docx-3-joh-10.1177_27551938241310120 for A Systematic Review of Evaluated Labor Market Initiatives Addressing Precarious Employment: Findings and Public Health Implications
