# Supplementary material for: A Systematic Review of Evaluated Labor Market Initiatives Addressing Precarious Employment: Findings and Public Health Implications
Source: Int J Soc Determinants Health Health Serv. 2025 Jan 15;55(3):268–88. doi: 10.1177/27551938241310120 (PMC12171052; doi:10.1177/27551938241310120)
Supplement: sj-docx-1-joh-10.1177_27551938241310120 - Supplemental material for A Systematic Review of Evaluated Labor Market Initiatives Addressing Precarious Employment: Findings and Public Health Implications [file sj-docx-1-joh-10.1177_27551938241310120.docx]

**Supplementary Material 1 - Systematic review methods**

**Sample search terms**

| Construct of interest | ((precari* or informal* or casual or atypical or non-standard or tempora* or gig or part-time or project-based or sub-contract) adj3 (work* or employ* or labour)) OR exp Employment |
| --- | --- |
| Intervention | (initiative* or intervention* or program* or strateg* or polic* or social security or legislation* or regulation* or legal provision* or directive* or labour standard* or organizational polic* or guideline* or recommendation* or collective agreement* or collective contract* or union agreement* or plan* or pilot or test* or trial* or experiment* or routine* or practice* or procedure* or childcare) or exp labor unions or exp social security or exp jurisprudence or exp child care or organizational policy |
| Evaluated initiatives | (evaluat* or assess* or apprais* or measure*) or program evaluation |

**Search strategies**

| Academic databases | PubMed, Scopus, andWeb of Science Core Collection |
| --- | --- |
| Sources of grey literature | The institutional databases of the International Labour Organization, European Foundation for the Improvement of Living and Working Conditions, and the Centers for Disease Control (CDC) and Prevention Community Guide of Evidence-Based Findings. |
| Other | Reviewed the reference lists of included studies, Conducted forward citation tracing, consulted relevant stakeholders for suggestions |

**Languages covered**

English abstract, and full text in any of these languages: Catalan, Danish, Dutch, French, Italian, Norwegian, Romanian, Spanish, and Swedish.

**Time period covered**

January 2000 to May 2021

**Data management**

Completed with the use of the following software: Covidence, Zotero, EndNote, Microsoft Teams, Google Forms, and Excel spreadsheets.

**Data selection and collection**

The title and abstract screening were completed independently by one reviewer, given the large number of records screened (#8150), after implementing several strategies to ensure decision-making consistency across reviewers. The strategies included clear guidance regarding inclusion/exclusion criteria, abstract screening training, pilot testing the screening process several times to ensure consistent understanding among all reviewers, and bi-weekly meetings to address questions. Studies indicated as Maybe were screened by two additional reviewers and conflicts resolved through discussion. Full-text screening was completed independently by two reviewers and conflicts resolved through discussions. For studies confirmed for inclusion, data extraction was completed independently by the two reviewers, using a tailored data extraction form to capture all relevant information about each study.

**Quality appraisal**

Because the eligible studies included a combination of qualitative, quantitative, and mixed methods designs, we used the Mixed Methods Appraisal Tool (MMAT) to assess the methodological quality of included studies. To make certain that the inclusion and exclusion criteria were applied consistently by the large team of 12 reviewers participating in the full-text review, as an added confirmatory step, a core team of four reviewers involved in the quality appraisal process, performed another full-text screening of all studies initially deemed eligible before conducting the qualitative assessment.
