# Supplementary material for: A Systematic Review of Evaluated Labor Market Initiatives Addressing Precarious Employment: Findings and Public Health Implications
Source: Int J Soc Determinants Health Health Serv. 2025 Jan 15;55(3):268–88. doi: 10.1177/27551938241310120 (PMC12171052; doi:10.1177/27551938241310120)
Supplement: sj-docx-2-joh-10.1177_27551938241310120 - Supplemental material for A Systematic Review of Evaluated Labor Market Initiatives Addressing Precarious Employment: Findings and Public Health Implications [file sj-docx-2-joh-10.1177_27551938241310120.docx]

**Identification of studies via other methods**

**Identification of studies via databases and registers**

Records identified from other sources: (n = 0)

Records removed *before screening*:

Duplicate records removed

(n = 3450)

Records removed for other reasons (n = 0)

Records identified from:

**Databases (n = 8475)**

PubMed (n=1769), Scopus n=3767)

Web of Science (n = 2939)

**Other sources (n = 3125)**

(gray lit, reference lists, citation tracing, stakeholder suggestions)

Registers (n = 0)

**Identification**

Records screened

(n = 8150)

Records excluded

(n = 7889)

Reports not retrieved

(n = 0)

Reports sought for retrieval

(n = 0)

Reports sought for retrieval

(n = 261)

Reports not retrieved

(n = 0)

**Screening**

Reports excluded:

Reason 1 (n = 0)

Reason 2 (n = 0)

Reason 3 (n = 0)

etc.

Reports assessed for eligibility

(n = 0)

Reports assessed for eligibility

(n = 261)

Reports excluded:212

Reason 1 (n = 23)

Reason 2 (n = 47)

Reason 3 (n = 30)

Reason 4 (n = 109)

Reason 5 (n = 2)

Reason 6 (n = 1)

Studies included in review (n=49)

Studies evaluating labour market initiatives discussed in this manuscript (n=22)

**Included**

**Supplementary Material 2. Flow diagram of study identification screening and inclusion**

Reasons for exclusion: **1**. Editorial, Commentary, Discussion Paper, Review; **2**. No clear initiative implemented; **3**. Initiative designed to facilitate PE or increase exposure to PE; Improve workers' health through individual behavioral change without a focus on PE; Improve work performance or health, safety, or well-being of workers with disabilities without a focus on PE; Eliminate or reduce workers’ exposure to unemployment; Eliminate, reduce, or mitigate the effects of unemployment on health and well-being; or Promote workers' return to work after illness or injury without addressing PE; **4**. Not evaluated formally or assessed using empirical data or The evaluation does not include a clear focus on the reduction of precarious employment and/or on precarious workers and/or their families. **5.** Duplicate. **6.** Not in a language mentioned in the protocol.
