# Supplementary material for: A Systematic Review of Evaluated Labor Market Initiatives Addressing Precarious Employment: Findings and Public Health Implications
Source: Int J Soc Determinants Health Health Serv. 2025 Jan 15;55(3):268–88. doi: 10.1177/27551938241310120 (PMC12171052; doi:10.1177/27551938241310120)
Supplement: sj-docx-3-joh-10.1177_27551938241310120 - Supplemental material for A Systematic Review of Evaluated Labor Market Initiatives Addressing Precarious Employment: Findings and Public Health Implications [file sj-docx-3-joh-10.1177_27551938241310120.docx]

Supplementary Material 3 - Countries examined, economic sector, population sub-groups, study objectives, implemented initiative, design, and data collection approaches used to evaluate initiatives

| **Study Author(s)**  **Publication Year**  **Countries Examined** | **Targeted Economic Sector**  **& Population Sub-groups** | **Study Objectives*** | **Implemented initiatives** | **Design and data collection/analysis approaches used to evaluate initiatives** |
| --- | --- | --- | --- | --- |
| **Labor market policies, legislation, and reforms** | | | | |
| (Fagernas, 2010)  India | Industrial, service, and agricultural sectors  All workers including the self-employed, regular salaried workers, and casual workers. | To analyze the relationship between labor regulation (to strengthen job security and the rights of workers and employers in industrial labor disputes) and the share of permanent salaried workers (used as proxy measure of formal employment) and temporary/casual and self-employed workers (used as proxy measure of informal employment because they are generally not covered by standard social security and job-security provisions, and many of the worker rights do not apply to them). | **Changes to industrial labor disputes legislation and the dispute settlement process adopted at state-level to strengthen the job security of workers and protect the rights of workers and employers in labor disputes**. Changes to industrial labor disputes legislation cover the functioning of labor courts, dispute settlement, and include revisions to the Industrial Disputes Act to increase protection of workers and employers, accelerate a pending dispute settlement process, and strengthen law enforcement. | Econometric analyses using fixed-effects regression models, involving within and across state comparisons. Pseudo-panel data set constructed with employment data for each individual state, obtained from four national cross-sectional household employment-unemployment surveys conducted by the National Sample Survey Organization with individual-level information. Data sources for context variables: Labor Bureau, Government of India, Ministry of Labor, Indian Labor Statistics. Industrial disputes legislation analyzed for the 1979 to 1999 period and outcomes measured in 1983, 1987, 1993, and 1999. |
| (Pla-Julián, 2014)  Spain | Domestic service sector  All workers | To assess the impact of law reforms concerning household employment on domestic work. | **Law reforms concerning household employment, aimed at improving the employment and working conditions of domestic workers.** Three key changes announced in 2021: (i) an agreement for employment growth and pension guarantee, focused on the reform of pensions to integrate the Special Regime for Household Employees into the overall social security system; (ii) a law aimed at revising and updating the social security system to gradually integrate the workers already covered by the Special Regime for Household Employees into the general social security system by 2018; and (iii) two Royal Decree-Laws regulating labor relations in households, with the goal of ensuring working conditions that protect the dignity of those providing domestic services. | Desk review and analysis of secondary data from statistics and coding/analysis/interpretation of primary interview data. Data sources: ILO statistical information; State Official Bulletin, National Statistical Institute; National Social Security Institute; Regional Immigration Office in Valencia (Spain), and Public Administration Secretariat; data from a qualitative study conducted in Valencia in 2012 and 2013, involving open ended in-depth interviews and focus groups with key informants and native and immigrant household workers. |
| (Arranz et al., 2013)  Spain | No specific sector  All workers, except for some initiatives targeted at young people under 29, over 45, individuals in long-term unemployment, women in certain situations, and persons with disabilities. | To examine if certain active labor market policies may incentivize permanent employment and job creation. | **Adoption of active labor market policies, such as employment subsidies for permanent contracts, job-creation schemes, and vocational training programs to fund/incentivize job creation and permanent employment at the country level over a period of 24 years.** Employment subsidies consist of several financial incentives provided to firms, such as reduced social security contributions for employers or being allowed to not follow the regulatory framework for regular contracts, to offer workers permanent contracts instead of temporary ones. Worker groups targeted included young people under 29, over 45, individuals in long-term unemployment, women in certain situations, and persons with disabilities. Job creation schemes involved the provision of grants to promote (i) self-employment and employment in cooperatives and employee-owned firms, (ii) promotion of local development and of firms/projects that prioritize employment, and (iii) participation in the labor market of persons with disabilities. Vocational training programs consisted of initiatives to expand and update workers’ skills and retrain individuals in unemployment. | Macroeconomic study using aggregate panel data for all 17 regions of Spain and 24 years (1987-2010), with the unit of analysis being a region. Labor market data drawn from the Active Population Survey and policy data from the Yearbook of Labor Statistics and Bulletin of Labor Statistics. Dynamic panel data model used to estimate the possible effect of different active labor market policies, including a generalized method-of-moments estimator to control for the possibility that the policy variables both affect and are affected by the dependent variables such as transitions to employment or permanent employment. Key limitation – difficult to separate the effects of active labor market policies from the effects of other public policies or factors impacting aggregate labor market outcomes. |
| (Mendez, 2013)  Spain | No specific sector  Temporary workers, especially youth less than 25 and workers over 45, men and women. | To analyze if two major labor market reforms were successful in reducing temporary employment and promoting permanent employment. | **Implementation of two major labor market reforms in Spain in 1994 and 1997 to reduce temporary employment and promote permanent employment.** The 1994 reform reinstated the ‘principle of causality in the application of temporary contracts’, introduced fiscal stimuli for the replacement of temporary contracts with permanent ones, lessened procedural obligations related to ‘fair’ firings, and shortened notice periods to decrease costs associated with permanent contracts. The 1997 reform, sanctioned by both trade unions and employer associations, revised the existing permanent contract stipulations to further reduce the costs associated with permanent contracts; it reduced the duration of severance pay for ‘unfair’ dismissals to 33 days of wages for each year of seniority and introduced payroll deductions of 40-80% for up to 2 years. The percentage and duration of tax reductions were higher for young workers and women and did not apply to middle-age unemployed workers. | Difference-in-difference estimation procedure that uses pre-treatment outcomes to predict post-treatment outcomes in the absence of the treatment (the 1994 and 1997 reforms). Data derived from a nationally representative survey, the Spanish Labor Force Survey, 1987 (2nd quarter) to 2000 (4th quarter). Certain groups were excluded: employers, the self-employed, agricultural, and family workers, co-op members, and those older than 65. |
| (Ciani and de Blasio, 2015)  Italy | No specific sector  Women and younger men (under 30). | To evaluate the effectiveness of using financial incentives to stimulate employers to convert fixed-term contracts into open-ended ones. | **The use of a national program offering incentives to stimulate employers to convert fixed-term contracts into open-ended ones.** The national program analyzed was based on a 2012 decree that provided financial subsidies to employers who (i) converted fixed term to open-ended contracts (12,000 euros per conversion); (ii) stabilized workers with non-standard contracts or who have been unemployed in the previous 6 months (12,000 euros per stabilization); and (iii) hired workers with a fixed-term contract to expand the existing workforce (3,000 to 6,000 euros depending on contract length). The program targeted women and younger men workers, excluding older men workers and mandated that the converted or stabilized job lasted for at least 6 months. | Difference-in-difference estimation strategy using 2012 administrative microdata for one region (Veneto), comparing different periods in 2012 and different groups.  Limitations: one region only, data gaps (e.g., info available re: eligible employees vs. employees using the subsidies; not all permanent and temporary contracts captured in the administrative microdata available) and limited data quality (e.g., self-reported and provided by employers). |
| (Borooah et al., 2007)  India | No specific sector  Own account workers, regular salaried or wage workers, casual wage laborers. | To study the effect of jobs reservation (affirmative action programs aimed at reserving certain proportion of government or public sector jobs for members of several castes and tribes) on improving the economic opportunities of individuals belonging to India’s Scheduled Castes (SC) and Scheduled Tribes (ST). | **Adoption of jobs reservation policies (affirmative action programs) to reserve a proportion of government or public sector jobs for persons from certain Scheduled Castes and Scheduled Tribes.** Job reservation policies, also referred to as compensatory discrimination in favor of those targeted by it, were adopted to improve access to ‘regular salaried or wage employment’* as opposed to being self-employed or casual laborers for persons from certain Scheduled Castes and Scheduled Tribes.  *Regular salaried or wage employment are viewed as being stable and secure, with decent income, meeting labor standards and providing social protection.(Fields, 2011) | Multinomial logit regression model used to assess the probability of a person with given characteristics being in one of the three employment categories: own account workers, regular salaried or wage workers, and casual wage laborers. Hindu and Muslim groups are used for comparison. Source: administrative employment data for the 1999-2000 period, extracted from the National Sample Survey. Key limitation – challenging to measure the impact of a policy on discrimination, since discrimination can be both caused by attributes of individuals (level of education) but can also cause these attributes (discrimination leads to poor education choices which leads to discrimination). Since assessing these indirect effects is not straightforward, the estimate of the effect of job reservations is an underestimate of discrimination. Job reservation policies might lead to less discrimination, which might lead to better choices by individuals, which might lead to better job outcomes. |
| (Giovannetti et al., 2021)  Egypt | Industry sectors whose output in terms of goods and services are traded internationally (traded sectors).  All workers | To assess the impact of tariff reforms on the labor market, with a focus on protecting wages and job stability, including having a permanent position. | **Adoption of tariff reforms** **(protectionist policies)** **to protect certain economic sectors from foreign competition.** The tariff reforms adopted by the government in 2016 raised import tariffs for 364 tariff lines, mostly non-agricultural goods, by 100 to 200% in most cases, with some increases varying between 50, 125, 300, 500, and 700%. Rationale: strong competition was linked to pressure on companies to cut production costs. The tariff reforms reversed previous decades of trade liberalization. | Econometric analysis using cross-sectional pooled OLS estimations. Data source: worker-level data from the available four waves of Egyptian Labor Market Panel Survey (1998, 2006, 2012, 2018. Limitations: the focus on traded sectors limits the generalizability of the findings to other sectors. |
| (Selwaness and Zaki, 2015)  Egypt | Manufacturing sector  Informal workers | To assess the effect of trade liberalization on informal employment. | **Adoption of trade liberalization reforms** **consisting of tariff reductions leading to lower trade costs (with potential to decrease informal employment).** While trade liberalization reforms could encompass diverse strategies, the focus of this study is on tariff reductions leading to lower trade costs. | Two-step regression analysis approach used: (i) the use of a probit model to estimate the probability of working in the informal sector in 1998 and 2006 and (ii) the pooling of industry coefficients over time (for 1998 and 2006) and their regression on tariffs. Data source: microeconomic data set (the Egyptian Labor Market Panel Survey) and macroeconomic variables (tariffs). Limitation: difficult to separate the impact of other macro-level economic factors, such as market flexibility, on informal employment. |
| (Rothenberg et al., 2016)  Indonesia | No specific sector  Informal workers | To test whether reducing business registration costs for firms decreases the share of informal businesses and the workers’ probability of being informally employed. | **Introduction of ‘one-stop shops’ for business registration, a large-scale program attempting to reduce registration costs for businesses, with the goal of reducing the number of informal businesses.** ‘One-stop-shops’ for business registration are programs endorsed, not mandated, through a national 2006 decree, provided by local governments in each district to allow the processing of main business licenses into one location, streamline the process, and lower the registration costs for firms. | Panel regression analyses using fixed effects models. Data source: 2010-2013 waves of the Survey of Micro and Small Enterprises (IMK). Limitation: data captured in the IMK survey covers the years 2010-2013 only, representing the end of the program, while its expansion years, 2006-2009, are not covered. To address this limitation, the 2000-2011 waves of the BPS’s national labor force household survey assessing the workforce were also used. |
| (Osorio-Copete, 2016)  Colombia | No specific sector  Informal workers | To evaluate the effectiveness of a tax reform in promoting labor formalization and reducing labor informality. | **Adoption of a tax reform lowering costs for firms with the goal of promoting labor formalization.** The reform consists of tax changes for firms resulting in (i) reduction of non-salary costs; (ii) changes in the ways in which profits are taxed; (iii) a new tax structure to tax the income of workers; and (iv) modification of tax rates for goods and services. | A mix of experimental analyses such as dynamic stochastic general equilibrium models and simulations using macroeconomic data exercises, followed by validation of findings with empirical data for 2012-2014. Data sources: Supply and Product Input matrices, the DANE National Account Balances, Parafiscal and Formal Employment report of the Ministry of Finance and Public Credit; OECD – Economic survey of Columbia. |
| **Union strategies** | | | | |
| (Wright, 2013)  UK | No specific sector  Unionized workers | To examine strategies used by unions to counteract the growth of precarious work through finding new ways to engage precarious workers who are not unionized. | **Adoption of two strategies (community unionism, and sustainable sourcing) by unions to reach precarious workers.** Community unionism aims to organize precarious workers outside of the workplace, based on the premise that traditional work­place-based strategies used by unions are not well suited for reaching and organizing workers in non-standard employment, given that their employment relationship is fragmented. In the context of fragmented production systems, sustainable sourcing strategies intend to counteract the negative effects resulting from pressure imposed by firms onto their suppliers through the use of procurement procedures and mechanisms that organize, extend collective bargaining, and improve conditions for workers in non-standard employment employed by suppliers and subcontractors. | Desk review of existing research examining successful initiatives used by unions. Data sources: union strategy documents and interviews with union officials. The success of strategies is assessed using a ‘strategic choice framework.’ Limitation: The evaluation is focused on process, not the actual impact of strategies on PE. |
| (Theodore, 2020)  US | Construction, Landscaping, Painting, Digging, Moving and Cleanup  Day workers, informal workers | To compare whether being employed for day labor through a worker center provides higher wages, more employment, and less instances of wage theft than being hired at informal hiring sites (e.g., street corners). | **The use of worker centers for the hiring of day labors instead of them being hired at informal hiring sites (such as street corners).** Worker centers act on behalf of the workers to regulate certain aspects of the informal economy through a range of regulatory actions, including recovering unpaid wages, advocating for public policies benefiting workers, initiating action campaigns against abusive employers, providing job and language skills, and training re: safe working practices, and community organizing. Worker centers build worker power through worker organizing and the strengthening of worker leaders. The worker center studied, Casa Latina in Seattle, Washington, DC, was established in 1994. It uses a hiring hall, a mean of regularizing the employment of day-labor through a hiring system that assigns jobs, establishes minimum wages, and increases transparency of employment arrangements to reduce wage theft. | Descriptive analysis based on worker surveys conducted in 2012 and 2015 to compare the wage rates, employment rates, and wage theft instances for workers hired through a worker center and those using four informal hiring sites. Limitation: small scale study of one worker center. |
| **Apprenticeships and/or other programs focused on youth and new graduates** | | | | |
| (Corseuil et al., 2019)  Brazil | No specific sector  Students graduating from secondary education institutions and youth workers | To investigate whether a large-scale apprenticeship program, consisting of special temporary contracts providing training to young workers, offers a better steppingstone to permanent jobs than other forms of temporary jobs. | **A national apprenticeship program, consisting of special temporary contracts providing classroom and skill training to young workers.** The national Brazilian Apprenticeship program aims to help young workers complete the transition to stable work through temporary contracts that provide both in-classroom training by certified institutions and concomitant on-the-job training, with a professional-skill certification provided at the end of the contract. The program is funded via payroll subsidies provided by the government to companies that opt to hire interested young workers through the special apprenticeship temporary contract. | Adjusted matching estimation using a matched employee-employer longitudinal dataset, RAIS - Relação Anual de Informações Sociais, covering all formal employees in Brazil, including apprentices. Control group: young workers with temporary contracts not included in the apprenticeship program. The analysis was done for youth enrolled in the program in the year 2000. Outcomes are measured for the short (2-3 years) and medium-term (4-5 years) after completing the program. |
| (Jackson and Collings, 2018)  Australia | No specific sector  Students graduating from university | To examine the benefit of various forms of practical experience on full-time employment and underemployment for students graduating from university. | **The completion of two types of practical experiences (unpaid work-integrated learning and paid work) by university students during their final year of studies.** The two types of practical experiences undertaken by university students in an Australian university assessed are: (i) one academic unit of work-integrated learning, a combination of academic and workplace learning, usually unpaid and (ii) paid work. | The employment outcomes of students who completed work-integrated learning and/or paid work during the final year of their studies and those who did not are compared. Data were collected in 2015 from students graduating in 2013, using two samples of domestic bachelor graduates from across disciplines, one obtained directly through a university survey, and one obtained from a national survey administered by universities. |
| (Ibarrarán et al., 2019)  Dominican Republic | No specific sector  Youth from disadvantaged socio-economic contexts | To assess the effects of a job-training program offered to disadvantaged youth on their employment opportunities and income. | **The offering of a paid job-training program consisting of classroom and on-the-job training along with an internship for disadvantaged youth.** The program evaluated, Juventud y Empleo/Youth and Employment, has been offered in the Dominican Republic since 2001 to 16 to 29-year-old youth who live in poor neighborhoods, do not attend school, did not complete high school, are underemployed / unemployed / or occupationally inactive at program registration. The program consists of 250 hours of classroom training and on-the job training for entry-level occupations, followed by a three-month internship in a private company. Participants receive a monetary pay of $3 USD per day and insurance against workplace injuries. | A large-scale randomized controlled trial of a youth training program, estimating effects six years after random assignment. Data collection done through three surveys collected through interviews in 2008 (baseline), 2010 and 2011 (18 and 24 months respectively after graduation), and 2014 (6 years after completing the program). |
| (Calero et al., 2017)  Brazil | No specified sector  Youth from disadvantaged socio-economic contexts | To assess if art-based interventions could improve labor market outcomes for youth. | **The provision of arts-based interventions (arts- and theater-based pedagogic tools) to youth from disadvantaged socio-economic contexts, part of a larger training program that includes vocational and academic training along with training in work-readiness skills.** The program is offered in Brazil since 2009 by a non-governmental organization aiming to increase various skills: vocational (e.g., construction-related skills, carpentry, soldering), work-readiness (e.g., finishing tasks, being punctual, communication), cognitive and socio-emotional skills (e.g., ability to persevere and follow through, ability to self-control) to youth who may lack them. The program is offered in a neutral downtown space, away from the violence of the favelas. It lasts approximately six months and provides training five days a week, for five hours daily. | Randomized controlled trial that started in 2012 and enrolled three cohorts, in April, June, and July. Three surveys (collected through interviews) were completed for each cohort, at baseline and two follow-up dates (at 2-5 months and at 11-13 months after program termination. |
| (Albanese et al., 2021)  Italy | No specified sector  Youth ≤ 29 years old | To analyze the effects of a reform to the apprenticeship system on labor market outcomes for apprentices. | **The reforming of a national apprenticeship system to facilitate stronger links between apprentices and employers and reduce bureaucracy facing companies offering the training.** The reform took place in 2003 and its implementation was staggered across regions and industries. The key reforms included (i) an increase in the legal length of an apprenticeship contract (from 1.5 -4 years previously to 2-6 years); (ii) the replacement of the previous requirement for formal in-school with on-the-job training; and (iii) the introduction of a minimum floor to apprentices’ wages. Companies offering apprenticeships benefit from payroll tax rebates and reductions in social security contributions. While the intent was for the reform to apply nationally, only certain regions implemented it. | Comparison between two groups of apprentices, one hired under the old rules and another under new rules. The analysis is done for a period of 7 years (longer than the average contract duration of 3-5 years), 2007 – 2014 and it involves covariance balancing scores to control for differences between the groups. Data source – administrative panel data derived from social security registers, covering different regions and industries. Sample consists of 17,948 individuals, 7,204 hired under the old system and 10,744 under the reformed one. Analysis not able to pinpoint the exact impact of each aspect of the reform on outcomes, but it is suggested that providing on-the-job training is a key determinant. |
| (Baumann et al., 2013)  Canada | Health sector  Nurse graduates (from registered nurse and registered practical nurse programs) | To assess the impact of a government policy funding full-time employment positions for up to six months for all new nursing graduates on securing full-time employment. | **The adoption of a governmental policy initiative** **to encourage full-time employment for new nurse graduates through the provision of funding (up to six months for salary & benefits) to employers hiring nurse graduates in full-time nursing positions, with the expectation that the full-time employment will be extended after the funded period.** The policy, called the Nursing Graduate Guarantee, was launched, and funded by the Ontario provincial government in 2007 in response to an increased casualization of the nursing workforce, especially affecting the new grads. The initiative covered graduates from Registered Nurse and Registered Practical Nurse programs completed in Ontario. Participating employers were expected to extend their full-time employment after 3-6 months unless there were outstanding reasons against it. If unable to meet this requirement within six months, employers had to commit to funding the new grad for at least an additional six-weeks of full-time employment. The expectation was that employers will also work on other strategies to increase the level of full-time employment. | Analysis period: 2012-2013. Mixed methods approach using primary and secondary data to compare full-time employment rates in new graduates who participated in the initiative and those who did not. Primary data included: (i) qualitative data from focus groups with healthcare employers, nurse graduates hired through the initiative, staff nurse mentors, and union representatives and (ii) quantitative data obtained through online surveys of healthcare employers, nurse graduates hired through this initiative, and union representatives. Secondary data extracted from the College of Nurses of Ontario and Ministry of Health and Long-Term Care databases covering new graduates. Sample: 6648 nurse graduates in 2012, of which 2249 participated in the initiative. |
| (Baumann et al., 2012)  Canada | Health sector  Nurse graduates (from registered nurse and registered practical nurse programs) | To assess the impact of a public policy initiative meant to stimulate full-time employment for new graduate nurses. | **The adoption of a governmental policy initiative** **to encourage full-time employment for new nurse graduates through the provision of funding (up to six months for salary + benefits) to employers hiring nurse graduates in full-time nursing positions, with the expectation that the full-time employment will be extended after the funded period.** The policy, called the Nursing Graduate Guarantee, was launched, and funded by the Ontario provincial government in 2007 in response to an increased casualization of the nursing workforce, especially affecting the new grads. The initiative covered graduates from Registered Nurse and Registered Practical Nurse programs completed in Ontario. Participating employers were expected to extend their full-time employment after 3-6 months unless there were outstanding reasons against it. If unable to meet this requirement within six months, employers had to commit to funding the new grad for at least an additional six-weeks of full-time employment. The expectation was that employers will also work on other strategies to increase the level of full-time employment. | Analysis period: 2006-2008. 2006 was considered the reference year. A ‘trend’ study design described as longitudinal research sampling the population of interest (nurse graduates) repeatedly was employed to assess the employment patterns of new graduate nurses over time. Quantitative survey data were extracted from the College of Nurses of Ontario database on graduate nurses, being complemented with a new graduate survey expanding on the College of Nurses of Ontario survey questions to learn about new graduate preferences for employment contracts (e.g., full-time, part-time, casual), sector of employment, and rural/urban. Sample: 2466 nursing graduates in 2006, 3152 in 2007, and 3175 in 2008. Limitations – new graduate nurse preference data re employment contract held versus contract desired was estimated using a convenience sample of nurses who answered the new graduate survey given that the nurse registration administrative database held by the College of Nurses of Ontario did not capture at the time whether a nurse has used the nurse graduate guarantee initiative and an individual’s preference for a certain employment contract. |
| **Social protection programs** | | | | |
| (Saavedra-Caballero and Londoño, 2018)  Colombia | No specified sector  Low-income and/or informally employed individuals and families with children aged 0 to 17. | To assess the impact of a conditional cash transfer provided to low-income and/or informally employed individuals and families with children aged 0 to 17 on labor market outcomes, such as probability to join the informal sector. | **Temporary provision of conditional cash transfers to low-income or informally employed individuals and families with children aged 0 to 17**. ‘Familias en Accion’ is a social assistance program offered across 691 municipalities by the Columbian government since 2001 with the goal of protecting and encouraging the formation of human capital among children from poor households, to alleviate the effects of the economic recession. Monthly cash transfer subsidies provided when certain conditions are met. Children < 7 years old had to be (i) vaccinated, (ii) attend health and nutritional check-ups, (iii) actively enrolled in school & attend at least 80% of classes, and their mothers had to attend health and nutrition presentations. | Quasi-experimental design across multiple time points (one and four years after program implementation), comparing labor market outcomes for parents who received the cash transfers and those who did not. Analysis uses propensity scoring and difference in difference estimation. Not clear what covariates lead to being selected in the treatment group. The sample (15,110 individuals and 7,267 households) size is quite large, but it is unclear if the survey used is a sample of families or if it consists of all families in the program. The ‘Familias en Accion’ dataset is used, as it provides pre-intervention and post-intervention (at 1 and 4 years) data. Limitations - Three definitions of informal employment are used (i) the legal standard definition (not enrolled in social security by employer), (ii) informality defined as non-salaried and having low education, and (iii) informality refers to anyone enrolled in the subsidized Columbian health system. |
| (Thornton et al., 2010)  Nicaragua | No specified sector  Informal workers and their families | To assess the impact of a subsidized voluntary health insurance program offered to informal sector workers on insurance program enrollment, health-related expenditures, outcomes, and patterns of utilization. | **Provision of a subsidized voluntary health insurance program to informal sector workers.** The program was initiated in 2007 by the government with the goal of improving access to and utilization of key health services and shielding families from high costs. The health insurance, previously offered only to formal sector employees, was extended to informal workers using three micro-finance institutions as delivery agents. | The success of the program was evaluated across a large sample of market vendors in one geographic region. The researchers randomly varied costs and convenience of enrolling to the program among the sample. Baseline and follow-up surveys were used to measure changes in health-care utilization and health. Focus groups and in-depth interviews were also conducted to increase understanding of decisions to enroll, stay enrolled, and utilize health services. Limitation – the evaluation of outcomes is done by comparing workers who were provided the insurance program at varied costs and via different providers rather than by comparing workers who were provided the insurance versus those who were not or by comparing workers before or after being provided the health insurance. |
| (Azuara and Marinescu, 2013)  Mexico | Not specified  Informal workers, self-employed workers, and individuals who are not economically active. | To assess the impact of universal health coverage provided to uninsured individuals on labor market informality, such as switching between the formal and informal sectors. | **Provision of public health coverage to uninsured workers, such as informal salaried workers and self-employed workers, and individuals not economically active.** The initiative reviewed is ‘Seguro Popular’, a health benefit program providing free health coverage to all those not insured by health institutions providing coverage for formal sector workers who make payroll contributions. | Quasi-experimental design, comparing data among municipalities that introduced the program at different times (progressive roll-out), using a balanced panel of municipalities that introduced the program in 2004 or earlier. Data sources: national and municipal census data and labor surveys, plus information about the roll-out of the program provided by the federal agency that coordinates the expansion of the program through the country. Panel regressions with municipality fixed effects. Period covered: 1995-2009. |

*Only the objectives most related to initiatives fitting the inclusion criteria for this review are listed.

References

Albanese, A., Cappellari, L., and Leonardi, M. (2021). The effects of youth labour market reforms: evidence from Italian apprenticeships. *Oxford Economic Papers, 73*(1), 98-121. doi:10.1093/oep/gpz053

Arranz, J. M., García Serrano, C., and Hernanz, V. (2013). Active labour market policies in Spain: A macroeconomic evaluation. *International Labour Review, 152*(2), 327-348. doi:10.1111/j.1564-913X.2013.00184.x

Azuara, O., and Marinescu, I. (2013). Informality and the expansion of social protection programs: Evidence from Mexico. *Journal of Health Economics, 32*(5), 938-950. doi:10.1016/j.jhealeco.2013.07.004

Baumann, A., Hunsberger, M., and Crea-Arsenio, M. (2012). Impact of public policy on nursing employment: Providing the evidence. *Canadian Public Policy, 38*(2), 167-179. doi:10.3138/cpp.38.2.167

Baumann, A., Hunsberger, M., and Crea-Arsenio, M. (2013). *Employment integration of nursing graduates: evaluation of a provincial policy strategy nursing graduate guarantee 2012-2013*. Retrieved from <https://books-scholarsportal-info.myaccess.library.utoronto.ca/en/read?id=/ebooks/ebooks0/gibson_cppc-chrc/2014-10-25/1/10901239#page=10>

Borooah, V. K., Dubey, A., and Iyer, S. (2007). The effectiveness of jobs reservation: Caste, religion and economic status in India. *Development and Change, 38*(3), 423-445. doi:10.1111/j.1467-7660.2007.00418.x

Calero, C., Diez, V. G., Soares, Y. S. D., Kluve, J., and Corseuil, C. H. (2017). Can arts-based interventions enhance labor market outcomes among youth? Evidence from a randomized trial in Rio de Janeiro. *Labour Economics, 45*(April), 131-142. doi:10.1016/j.labeco.2016.11.008

Ciani, E., and de Blasio, G. (2015). Getting stable: an evaluation of the incentives for permanent contracts in Italy. *IZA Journal of European Labor Studies, 4*(1), 1-6. doi:10.1186/s40174-015-0030-5

Corseuil, C. H., Foguel, M. N., and Gonzaga, G. (2019). Apprenticeship as a stepping stone to better jobs: Evidence from Brazilian matched employer-employee data. *Labour Economics, 57*, 177-194. doi:10.1016/j.labeco.2019.02.002

Fagernas, S. (2010). Labor law, judicial efficiency, and informal employment in India. *Journal of Empirical Legal Studies, 7*(2), 282-321. doi:10.1111/j.1740-1461.2010.01179.x

Fields, G. S. (2011). Labor market analysis for developing countries. *Labour economics, 18*(1), S16-S22. doi:10.1016/j.labeco.2011.09.005

Giovannetti, G., Marvasi, E., and Vivoli, A. (2021). The asymmetric effects of 20 years of tariff reforms on Egyptian workers. *Economia Politica, 38*(1), 89-130. doi:10.1007/s40888-020-00199-0

Ibarrarán, P., Kluve, J., Ripani, L., and Rosas Shady, D. (2019). Experimental evidence on the long-term effects of a youth training program. *ILR Review, 72*(1), 185-222. doi:10.1177/0019793918768260

Jackson, D., and Collings, D. (2018). The influence of work-integrated learning and paid work during studies on graduate employment and underemployment. *Higher Education, 76*(3), 403-425. doi:10.1007/s10734-017-0216-z

Mendez, I. (2013). Promoting permanent employment: lessons from Spain. *Journal of the Spanish Economic Association, 4*(2), 175-199. doi:10.1007/s13209-012-0088-5

Osorio-Copete, L. M. (2016). Tax reform and labor informality in Colombia: A dynamic and stochastic general equilibrium analysis. *Ensayos Sobre Politica Economica, 34*(80), 126-145. doi:10.1016/j.espe.2016.03.005

Pla-Julián, I. (2014). Addressing informality, gender and ethnicity in domestic labour: lessons from recent Spanish legislation. *European Review of Labour and Research, 20*(4), 559-575. doi:10.1177/1024258914550777

Rothenberg, A. D., Gaduh, A., Burger, N. E., Chazali, C., Tjandraningsih, I., Radikun, R., . . . Weilant, S. (2016). Rethinking Indonesia’s informal sector. *World Development, 80*, 96-113. doi:10.1016/j.worlddev.2015.11.005

Saavedra-Caballero, F., and Londoño, M. O. (2018). Social assistance and informality: Examining the link in Colombia [Assistência social e informalidade: Analisando a relação na Colômbia] [Asistencia social e informalidad: Analizando la relación en Colombia]. *Revista de Economia del Rosario, 1*(81-120).

Selwaness, I., and Zaki, C. (2015). Assessing the impact of trade reforms on informal employment in Egypt. *Journal of North African Studies, 20*(3), 391-414. doi:10.1080/13629387.2014.997717

Theodore, N. (2020). Regulating informality: Worker centers and collective action in day‐labor markets. *Growth and Change, 51*(1), 144-160. doi:10.1111/grow.12343

Thornton, R. L., Hatt, L. E., Field, E. M., Islam, M., Solís Diaz, F., and González, M. A. (2010). Social security health insurance for the informal sector in Nicaragua: a randomized evaluation. *Health Economics, 19*(S1), 181-206. doi:10.1002/hec.1635

Wright, C., F. . (2013). The response of unions to the rise of precarious work in Britain. *The Economic and Labour Relations Review, 24*(3), 279-296. doi:10.3316/ielapa.746029458785026
